# Predictive value of preoperative esophagram in postoperative outcomes after antireflux surgery

**DOI:** 10.1007/s00464-026-13126-4

**Published:** 2026-07-31

**Authors:** Adam De Jesus, Brooklynn Rylee Rickett, Matthew Smith, Sean Phillip Duminie, David Harris, Amber Shada, Anne Lidor

**Affiliations:** Department of Surgery, Health Sciences Learning Center, University of Wisconsin School of Medicine and Public Health, University of Wisconsin, 750 Highland Ave, Madison, WI 53726 USA

**Keywords:** Esophagram, Gastroesophageal reflux disease, Nissen fundoplication, Esophageal transit time, Hiatal hernia, Manometry

## Abstract

**Background:**

Gastroesophageal reflux disease (GERD) significantly impairs quality of life. Antireflux surgery (ARS) improves symptoms when medical therapy fails, but postoperative recurrence and dysphagia remain concerns, emphasizing the need for improved preoperative risk stratification. This study evaluates whether esophageal transit time (ETT) measured on standard preoperative esophagram can predict postoperative outcomes after ARS and serve as an accessible method to characterize esophageal motility on esophagram and assist in preoperative decision-making.

**Methods:**

In this retrospective cohort study, 174 patients underwent Nissen or Toupet fundoplication (± hiatal hernia repair) between 2014–2025 at a tertiary academic center. Patients were required to have preoperative esophagram and ≥ 1 postoperative Health-Related Quality of Life (HRQL) follow-up; 92 patients also had high-resolution manometry. Preoperative esophagrams were independently reviewed by a subspecialized radiologist blinded to clinical outcomes. ETT was correlated with manometric findings and postoperative HRQL scores.

**Results:**

Receiver Operating Characteristic (ROC) analysis identified ETT threshold ≥ 5.375 s as associated with greater long-term symptom burden across all patient-reported outcome domains at 3–5 years (*p* < 0.05). Longer ETT was associated with more failed contractions and weaker distal esophageal contractions on manometry, and ETT showed sensitivity of 75–83%, specificity of 35–46%, positive predictive value (PPV) of 15–55%, negative predictive value (NPV) of 71–94%, positive likelihood ratio (LR +) (1.22–1.54), and negative likelihood ratio (LR-) (0.37–0.65).

**Conclusion:**

ETT greater than 5.375 s was associated with greater long-term symptom burden across all patient-reported outcome domains. Longer ETT correlated with higher percentages of failed contractions and lower distal esophageal contraction amplitude on manometry supporting physiologic relevance. While specificity is low, high NPV suggests ETT less than 5.375 s may serve as a simple, noninvasive screening to help exclude severe dysmotility and reduce reliance on manometry in selected patients. These findings support using ETT as an adjunctive risk-stratification measure to assist in preoperative decision-making rather than a standalone prognostic test.

**Supplementary Information:**

The online version contains supplementary material available at https://doi.org/10.1007/s00464-026-13126-4.

Gastroesophageal reflux disease (GERD) is a highly prevalent condition that significantly impairs quality of life, affecting an estimated 10–20% of the population and more than 75–150 million individuals in Europe and the United States [1]. Symptoms like heartburn, regurgitation, chest pain, and dysphagia can be life-altering [2]. Beyond symptom burden, chronic acid exposure may lead to long-term esophageal injury, including erosive esophagitis, Barrett’s esophagus, strictures, and adenocarcinoma [3].

Antireflux surgery (ARS), including Nissen and Toupet fundoplication with or without hiatal hernia repair, mechanically reinforces the lower esophageal sphincter (LES) to reduce pathologic gastroesophageal reflux and serves as an important alternative to medical management for appropriately selected patients [4, 5]. Current clinical guidelines support ARS for patients with medically refractory GERD or those who prefer surgical intervention [5, 6]. However, a substantial subset of patients continues to experience suboptimal outcomes, including persistent or recurrent GERD, dysphagia, gas bloat symptoms, continued need for proton pump inhibitors, or the need for additional medical or surgical therapy like repeat imaging studies, esophageal dilation, and repeat operation [4, 7, 8]. These varied postoperative courses highlight the need for improved preoperative risk stratification to identify patients most likely to benefit from ARS.

Currently, there is no unanimous consensus established to guide preoperative workup while ensuring optimal postoperative outcomes following ARS. Although current recommendations include esophagogastroduodenoscopy (EGD), manometry, and pH testing in the preoperative evaluation of GERD, the most recent multi-society SAGES guideline noted in Key Question 1 that, while manometry may be helpful, a formal GRADE recommendation could not be made because of insufficient evidence [9]. High-resolution manometry (HRM) provides a detailed assessment of esophageal motility and categorizes dysfunction according to the Chicago Classification, including disorders of esophagogastric junction (EGJ) outflow and peristalsis [10]. HRM is routinely performed before ARS to exclude achalasia and other motility disorders that may worsen following surgery [11]. Despite its routine use, the utility and necessity of routine HRM remain debated, given the cost, patient discomfort, and limited impact on surgical planning when the esophagram is normal [12]. Some investigators argue that HRM should not be mandatory before ARS [13–15]. Several studies indicate that no HRM parameter reliably predicts postoperative symptom recurrence or the need for reintervention [16, 17].

In contrast, the barium esophagram is a non-invasive, inexpensive, and safe method which provides structural and functional information, enabling dynamic visualization of bolus transit, hiatal hernia anatomy, and the gastroesophageal junction (GEJ) location, while also aiding in the detection of motility disorders such as achalasia and distal esophageal spasm [18]. Esophageal transit time (ETT), the duration required for a swallowed bolus to pass from the first rib to the gastroesophageal junction, quantifies the effectiveness of esophageal peristalsis and clearance function, enabling assessment of esophageal motility [19, 20]. Multiple studies have shown that prolonged ETT is associated with esophageal dysmotility and correlates with clinical symptoms. Patients with motor disorders such as achalasia, diffuse esophageal spasm, and scleroderma demonstrate significantly delayed transit [21]. Current SAGES recommendations do not include esophagram in the preoperative evaluation of GERD, but studies have found that when patients present with preoperative dysphagia and have delayed esophageal transit on barium esophagram, it is prognostic of poor postoperative outcomes [16, 22]. Overall, inconsistent radiographic definitions, variable recommendations, and term-to-term validation have been lacking, reinforcing our stance that the workup for patients with surgical GERD has many unanswered questions [9]. Measuring with a standard-view esophagram may offer a practical, reproducible metric for assessing motility and identifying patients at increased risk of postoperative complications. Therefore, the purpose of this study is to determine whether ETT correlates with HRM findings and predicts postoperative outcomes— measured by Health-Related Quality of Life (HRQL) questionnaire scores—following ARS, with the aim to reduce reliance on preoperative esophageal manometry if ETT is normal.

## Material and methods

### Patient population

This study is a retrospective cohort study of a prospectively maintained database of all adult patients who underwent Nissen or Toupet fundoplication, with or without concurrent hiatal hernia repair, at the University of Wisconsin between 2014 and 2025. Patients were eligible for inclusion if they underwent a preoperative esophagram and completed at least one postoperative HRQL assessment at 6 weeks, 1 year, 3 years, or 5 years. All patients considered for antireflux surgery have an esophagram performed routinely. Fewer patients had HRQL data available at longer follow-up, so the 3– and 5–year time points were combined, and for each patient, the most distant available HRQL assessment was used. Postoperative reinterventions were also assessed through the most distant available follow-up time point and included any reintervention, endoscopic dilation, reoperation, and repeat imaging. A subset of patients who had also undergone preoperative esophageal manometry was analyzed separately. Patients were excluded if they had a diagnosis of achalasia or had previously undergone operative foregut surgery All patient data were de‑identified and stored securely in REDCap (Research Electronic Data Capture) to ensure confidentiality. The study was approved by the University of Wisconsin Institutional Review Board (IRB) which included a waiver of informed consent.

### Symptoms assessment

Patient‑reported outcomes were evaluated using the Gastroesophageal Reflux Disease Health-Related. Quality of Life (GERD–HRQL) questionnaire, Eckardt score, Gastroparesis Cardinal Symptom Index (GCSI), and Reflux Symptom Index (RSI), all validated tools for assessing symptom changes following medical or surgical therapy. The GERD–HRQL instrument quantifies typical GERD‑related symptoms— including heartburn, dysphagia, odynophagia, gas bloat—as well as the impact of medications and overall patient satisfaction [23]. Each item is rated on a six‑point Likert scale: 0 = no symptoms; 1 = noticeable but not bothersome; 2 = noticeable and bothersome but not daily; 3 = bothersome daily; 4 = symptoms interfere with daily activities; and 5 = symptoms are incapacitating. A final question evaluates overall condition satisfaction and is categorized as “satisfied,” “neutral,” or “dissatisfied.” Total scores range from 0 (no symptoms) to 50 (severe symptoms).

The Eckardt score measures achalasia severity across four domains: dysphagia, regurgitation, chest pain, and weight loss [24]. Symptom frequency for the first three components is scored from 0 to 3, where 0 indicates absence, 1 occasional symptom, 2 daily symptoms, and 3 symptoms with every meal. Weight loss is scored as 0 for none, 1 for < 5 kg, 2 for 5–10 kg, and 3 for > 10 kg. Total scores range from 0 to 12, with values < 3 indicating treatment success and values > 3 suggesting suboptimal outcomes.

GCSI measures gastroparesis severity across three subscales: post-prandial fullness/early satiety (4 items); nausea/vomiting (3 items), and bloating (2 items). Each item is rated on a six‑point Likert scale: 0 = no symptoms; 1 = noticeable but not bothersome; 2 = noticeable and bothersome but not daily; 3 = bothersome daily; 4 = symptoms interfere with daily activities; and 5 = symptoms are incapacitating [25].

RSI is a validated patient-reported questionnaire used to assess laryngopharyngeal and extra-esophageal reflux symptoms, including hoarseness, throat clearing, excess throat mucus or postnasal drip, dysphagia, cough, globus sensation, and reflux-related throat or chest symptoms. Each item is scored from 0 to 5, with total scores ranging from 0 to 45; higher scores indicate greater symptom burden, and scores > 13 are generally considered abnormal in validation studies [26].

### Radiographic methods

Radiological assessment of preoperative esophagram studies was retrospectively re-evaluated by two radiologists blinded to patient information and outcomes. The motility series is one of the most important standard components of the esophagram and allows assessment of the esophageal function, performed with a single swallow in the prone oblique position to avoid gravity assist. A common protocol includes ingestion of a low density barium suspension during high frame rate fluoroscopy (7.5–15 frames/second). The motility series was identified, and the following equation calculated the ETT:

$${\mathrm{ETT}}\left( {\mathrm{s}} \right){ = }\frac{{\text{frame inlet - frame LES}}}{{{\mathrm{FPS}}}}$$

where frame_inlet_ is the frame # when the superior edge of the bolus reached the first rib (thoracic inlet), frame_LES_ is the frame # when the superior edge of the bolus reached the LES. FPS is the frame rate, recorded from the DICOM header (Supplemental Fig. 1). Studies were excluded from analysis if they were missing a motility cine or performed an insufficient protocol for the motility assessment (ex. more than a single swallow, non-visualization of the LES).

**Fig. 1:**
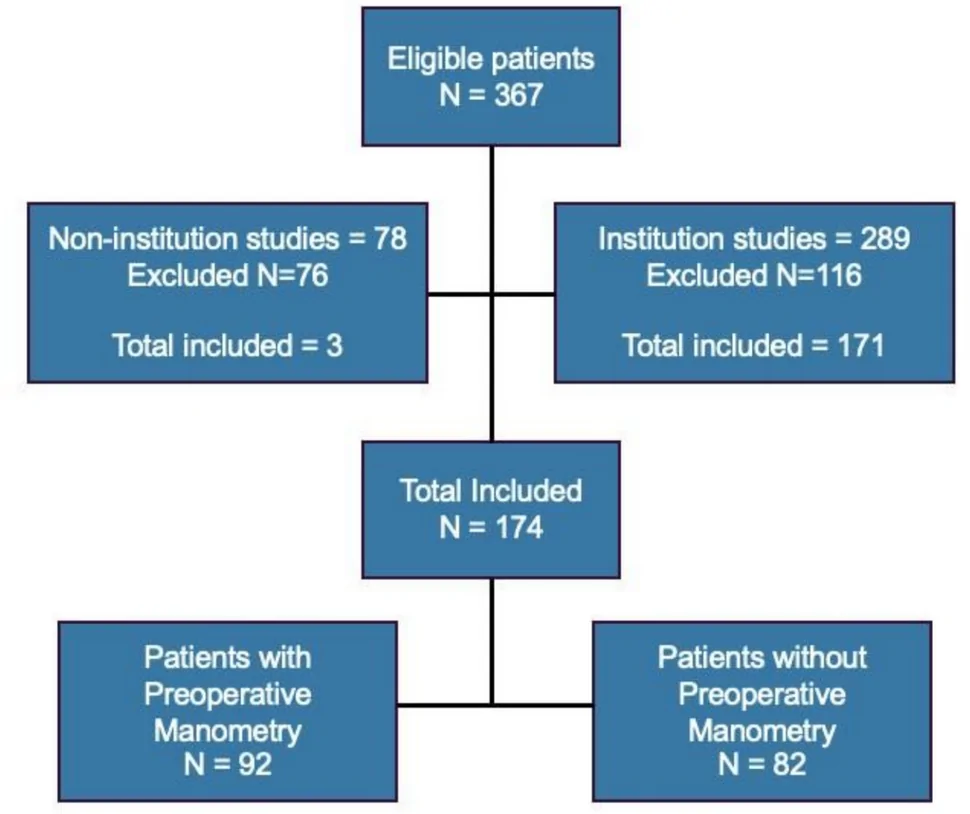
Cohort selection flow diagram. Patients were screened for inclusion from the institutional cohort of individuals undergoing antireflux surgery between 2014 and 2025. Exclusions included outside institution studies without interpretable motility imaging, missing motility data, non-visualization of the LES, premature termination of the fluoroscopic video sequence, late cine start time, prior foregut surgery, achalasia, and studies with multiple swallows. The final analytic cohort included patients with usable preoperative esophagram studies, with a subset also having preoperative manometry available for correlation analyses

### Statistical methods

Analyses were performed in a Julius AI-assisted Python environment and reviewed by the authors with University of Wisconsin statistical support. Continuous variables were assessed for normality using the Shapiro–Wilk test and summarized as mean ± standard deviation or median [interquartile range], as appropriate. Group comparisons were performed using Welch t-tests or Mann–Whitney *U* tests for continuous variables and chi-square tests for categorical variables. Correlations were assessed using Pearson correlation coefficients with 95% confidence intervals, and receiver operating characteristic analyses were used to evaluate candidate ETT cutoffs across long-term symptom outcomes. Missing data were handled by complete-case analysis without imputation, all tests were two-sided, and statistical significance was defined as *p* < 0.05. [27]. This manuscript was prepared in accordance with the STROBE Statement to promote transparent reporting of this retrospective cohort study [28].

## Results

### Cohort assembly and baseline characteristics

A total of 367 patients were eligible for review. Of these, 78 underwent outside-institution esophagram studies, and 76 of those were excluded because the studies lacked appropriate functional motility imaging. Among 289 in-institution studies, 116 were excluded because of missing motility data, non-visualization of the LES to calculate ETT, premature termination of the fluoroscopic video sequence, or late cine start time. The final analytic cohort therefore included 174 patients, of whom 92 had preoperative manometry available and 82 did not (Fig. 1). The cohort had a mean age of 58.7 ± 14.4 years and mean BMI of 30.5 ± 4.6 kg/m^2^. Most patients were female and underwent minimally invasive Toupet or Nissen fundoplication, usually with concurrent hiatal hernia repair. Detailed demographic, clinical, and operative characteristics are summarized in Table 1.

**Table 1:** Baseline demographic, clinical, and operative characteristics of the analytic cohort

Variable	Overall (*N* = 174)
Demographics	
Age (years)	58.7 ± 14.3
Female	122 (70.1%)
Male	52 (29.9%)
BMI (Kg/m^2)^	30.5 ± 4.6
Weight at initial visit (lbs)	190.7 ± 35.9
Race	
Black or African American	3 (1.7%)
White	168 (96.6%)
Hispanic or Latinx	3 (1.7%)
Primary reason for evaluation	
Reflux	134 (77.0%)
Dysphagia	17 (9.8%)
pain	30 (17.2%)
Preoperative symptoms	
Nausea	70 (40.2%)
Bloating	85 (48.9%)
Chest pain	87 (50.0%)
Diarrhea	27 (15.5%)
Flatulence	36 (20.7%)
Vomiting	54 (31.0%)
Dyspnea	55 (31.6%)
Dysphagia	91 (52.3%)
Abdominal pain	59 (33.9%)
Reflux	164 (94.3%)
Operative details	
Nissen fundoplication	72 (41.4%)
Toupet fundoplication	91 (52.3%)
Hiatal hernia repair	166 (95.4%)
Hiatal hernia type	
Type 1	62 (35.6%)
Type 2	4 (2.3%)
Type 3	78 (44.8%)
Type 4	4 (2.3%)
Laparoscopic approach	161 (92.5%)
Robotic approach	14 (8.0%)
Endoscopic approach	1 (0.6%)

Summary table of age, body mass index, sex, race, presenting symptoms, operative approach, fundoplication type, and hiatal hernia characteristics for included patients. Values are presented as mean ± SD or *n* (%)

### Baseline demographic and clinical characteristics stratified by ETT

When stratified by ETT threshold, 113 patients (64.9%) had ETT ≥ 5.375 s and 61 patients (35.1%) had ETT < 5.375 s. Baseline demographic and operative characteristics were broadly similar between groups, including age, sex distribution, BMI, weight, operative approach, and fundoplication type. The main differences were dyspnea, which was more frequent in patients with ETT ≥ 5.375 s, and hiatal hernia type distribution, with type I hernias more common in the prolonged ETT group and type III/IV hernias more common in the shorter ETT group. Detailed ETT-stratified baseline characteristics are shown in Supplemental Fig. 3.

### ROC analysis identified esophageal transit time thresholds associated with long-term symptom burden

**Fig. 2:**
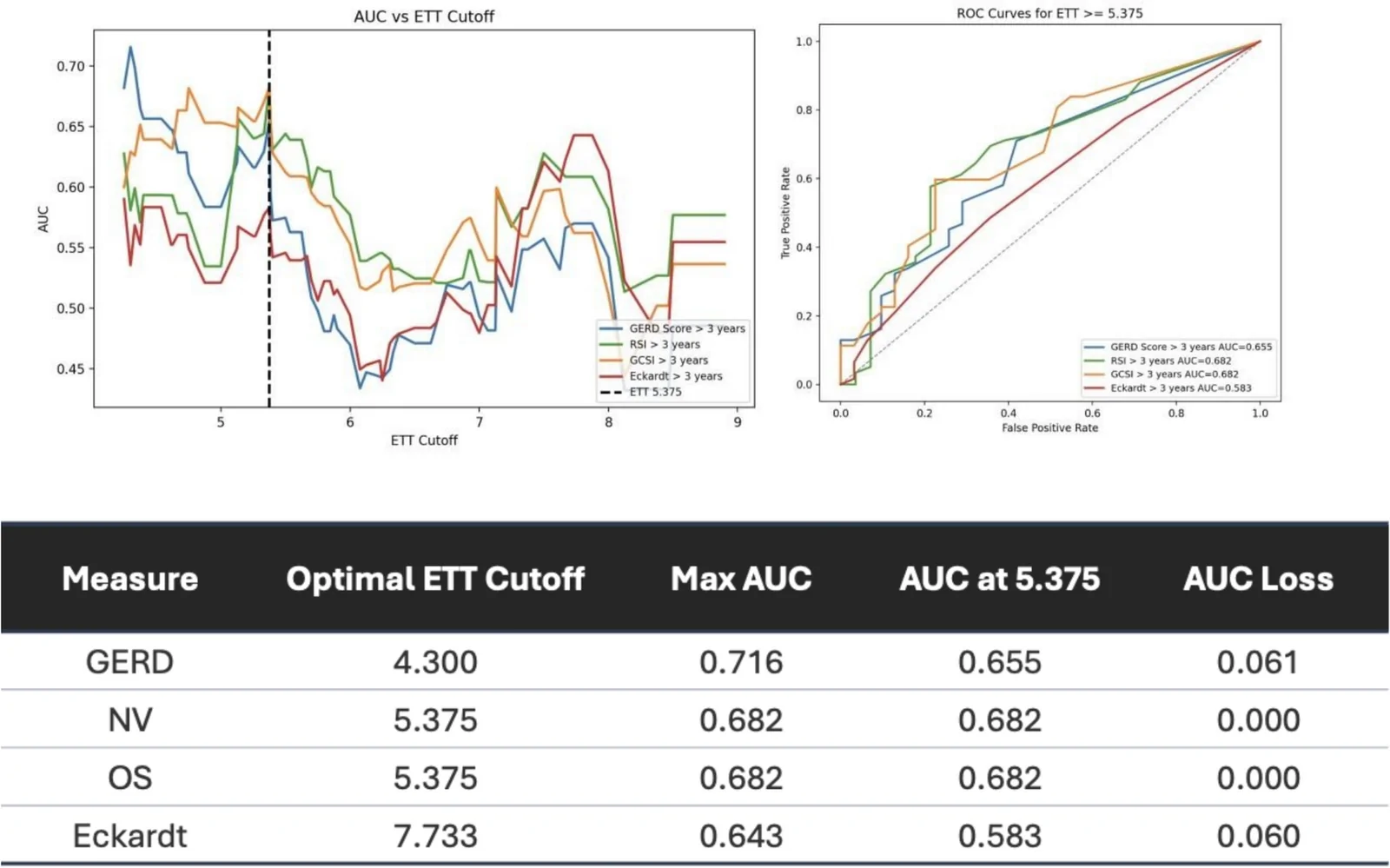
ROC analysis of preoperative esophageal transit time (ETT) versus dichotomized long-term outcomes (> 3-year scores, using the furthest available follow-up: 5-year if available, otherwise 3-year). Optimal exploratory cutoffs were identified by Youden index; the publication threshold of 5.375 s was retained for consistency across domains

ROC analysis was used to identify candidate ETT thresholds associated with worse long-term postoperative symptom burden. Although the optimal cutoffs varied across symptom domains, 5.375 s performed consistently across multiple outcomes and was selected as the exploratory threshold for subsequent analyses (Fig. 2).

### Postoperative symptom scores stratified by preoperative ETT

**Fig. 3:**
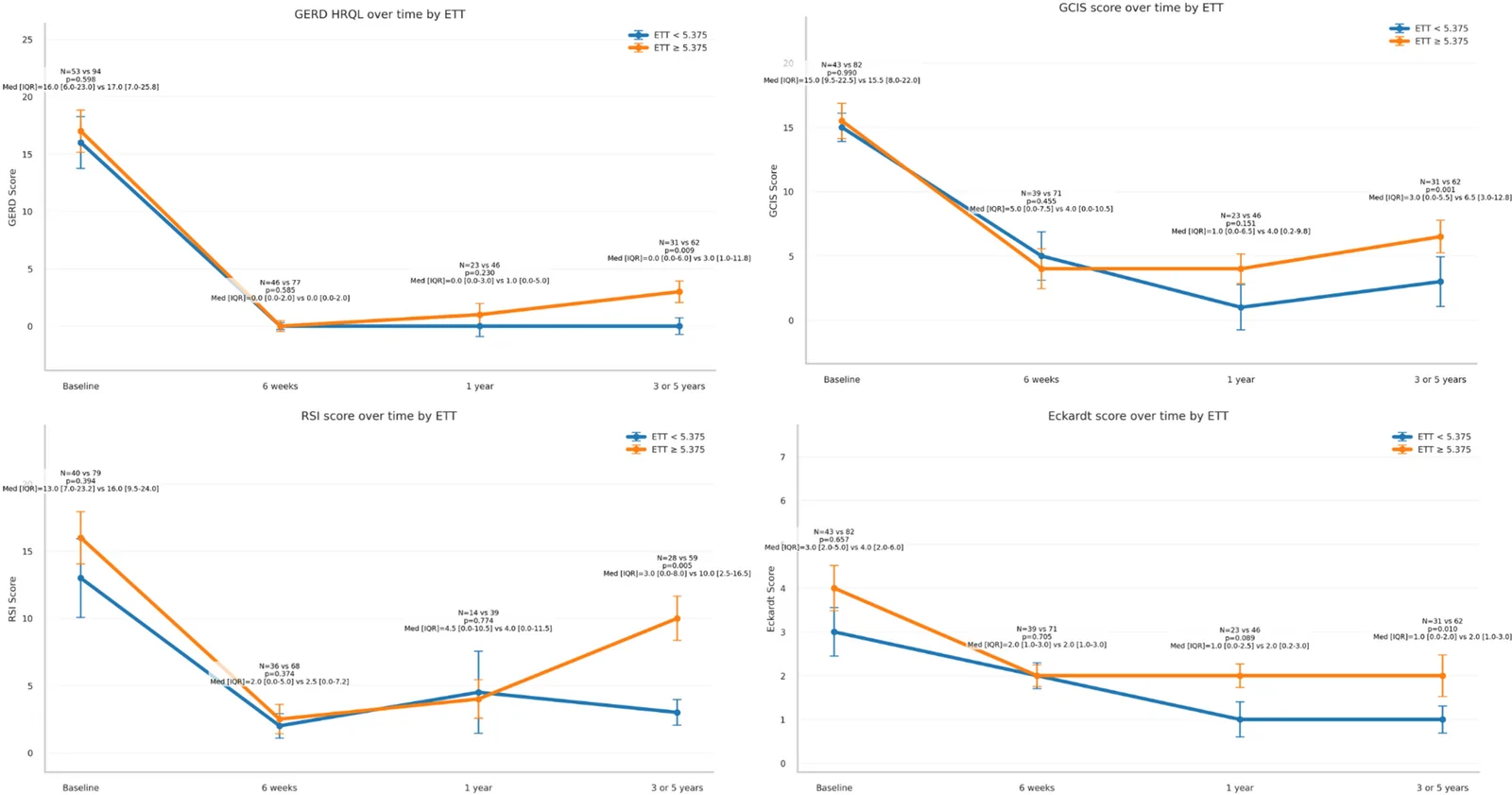
Longitudinal GERD–HRQL, GCSI, RSI, and Eckardt scores are shown for patients with ETT < 5.375 s versus ≥ 5.375 s at baseline, 6 weeks, 1 year, and long-term follow-up. Symptom scores were non-normally distributed, values are presented as median [IQR], and between-group comparisons were performed using two-sided Mann–Whitney *U* tests. For long-term outcomes, the furthest available follow-up score was used for each patient, with 5-year data used when available and 3-year data used otherwise. No multivariable adjustment or multiple comparison correction was performed for these longitudinal comparisons

When patients were stratified by preoperative ETT < 5.375 s versus ≥ 5.375 s, symptom scores were similar between groups at baseline, 6 weeks, and 1 year across GERD–HRQL, GCSI, RSI, and Eckardt domains. In contrast, group separation became more apparent at the furthest available long-term follow-up time point, defined as the 5-year value when available and otherwise the 3-year value. At long-term follow-up, patients with ETT ≥ 5.375 s had significantly greater symptom burden across all patient-reported outcome domains, including GERD–HRQL, GCSI, RSI, and Eckardt scores (Fig. 3, Supplemental Table 6). At long-term follow-up, patients with ETT ≥ 5.375 s had significantly greater symptom burden than those with ETT < 5.375 s across all patient-reported outcome domains, including GERD–HRQL (3.0 [1.0–11.8] vs 0.0 [0.0–6.0], *p* = 0.009), GCSI (6.5 [3.0–12.8] vs 3.0 [0.0–5.5], *p* = 0.001), RSI (10.0 [2.5–16.5] vs 3.0 [0.0–8.0], *p* = 0.005), and Eckardt score (2.0 [1.0–3.0] vs 1.0 [0.0–2.0], *p* = 0.010) (Fig. 3).

### Correlation analyses between ETT, manometric variables, and symptom scores

**Fig. 4:**
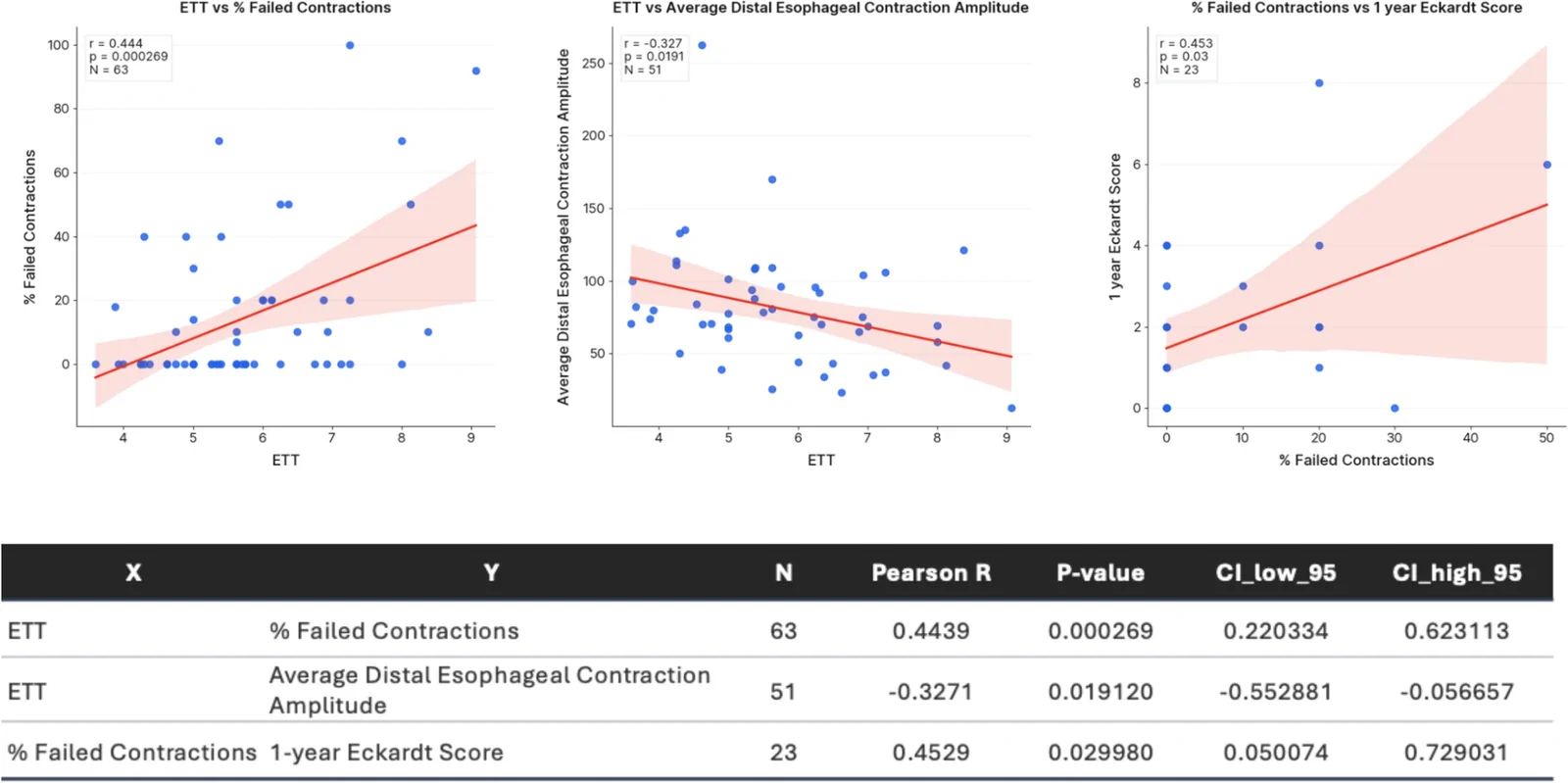
Correlation of esophageal transit time with manometric parameters and early postoperative symptom burden. Scatterplots demonstrate associations between preoperative ETT and percent failed contractions, ETT and average distal esophageal contraction amplitude, and percent failed contractions and 1-year Eckardt score. Longer ETT was positively correlated with a higher percentage of failed contractions, while longer ETT was negatively correlated with average distal esophageal contraction amplitude. In addition, a greater percentage of failed contractions was associated with worse 1-year Eckardt score. Scatterplots show unadjusted Pearson correlations with two-sided p values and 95% confidence intervals for r. Regression lines are descriptive only

Among patients with available manometric data, longer preoperative ETT was associated with markers of impaired esophageal motility. Specifically, longer ETT correlated with a higher percentage of failed contractions and lower average distal esophageal contraction amplitude (Fig. 4; Supplemental Fig. 2). The strongest associations were between ETT and percent failed contractions (r = 0.444, R^2^ = 0.197, *n* = 63), ETT and average distal esophageal contraction amplitude (r = –0.327, R^2^ = 0.107, *n* = 51), percent failed contractions and 1-year Eckardt score (r = 0.453, R^2^ = 0.205, *n* = 23), and percent simultaneous contractions and 1-year Eckardt score (r = 0.48, R^2^ = 0.227, *n* = 18). Although these relationships were statistically significant, the correlation coefficients and R^2^ values were modest, indicating that ETT and manometric variables capture related physiology but remain limited predictors of individual patient outcomes (Fig. 4).

### Test characteristics of manometry and esophagram for long-term symptom outcomes

**Fig. 5:**
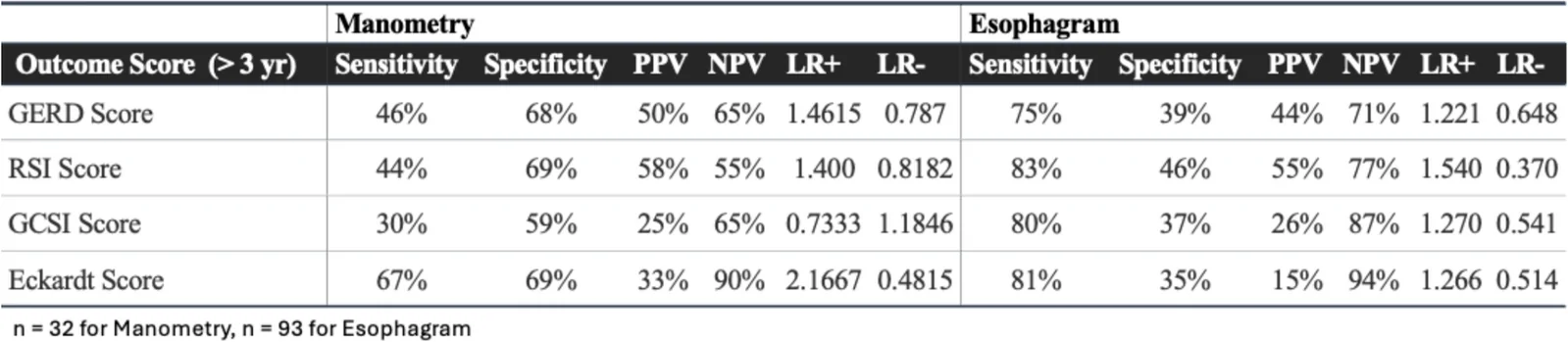
Test characteristics of manometry and esophagram for long-term symptom outcomes. Diagnostic performance measures, including sensitivity, specificity, positive predictive value, negative predictive value, and positive and negative likelihood ratios, are shown for percent failed contractions on manometry and esophageal transit time on esophagram in predicting worse long-term postoperative symptom outcomes across GERD, RSI, GCSI, and Eckardt domains

Test characteristics for poor long-term postoperative symptom outcomes were compared between percent failed contractions on manometry and ETT on esophagram using the 5.375-s threshold. Overall, ETT demonstrated higher sensitivity across symptom domains, ranging from 75–83%, compared with 30–67% for manometry, while manometry generally demonstrated higher specificity, ranging from 59–69%, compared with 35–46% for ETT. The negative predictive value of ETT was highest for GCSI and Eckardt outcomes, reaching 87% and 94%, respectively, suggesting that shorter ETT may be more useful for identifying patients less likely to develop poor long-term symptom burden. However, ETT had limited specificity and modest positive likelihood ratios across outcomes (LR + 1.22–1.54), indicating limited utility as a standalone rule-in test. Taken together, these findings support ETT as a potential screening adjunct rather than a replacement for manometry or an independent prognostic test (Fig. 5).

### Preoperative ETT was not associated with reintervention

Preoperative ETT was not significantly associated with postoperative reintervention, including any reintervention, endoscopic dilation, reoperation, or repeat imaging. Mean ETT was similar between patients who did and did not undergo any reintervention (6.09 vs 6.05 s, *p* = 0.509), endoscopic dilation (5.96 vs 6.07 s, *p* = 0.965), reoperation (6.89 vs 6.03 s, *p* = 0.150), or repeat imaging (6.02 vs 6.09 s, *p* = 0.848). Although patients requiring reoperation had numerically longer ETT, this difference was not statistically significant. These findings suggest that ETT was more strongly associated with long-term patient-reported symptom burden than with downstream procedural outcomes in this cohort (Supplemental Fig. 4).

### Postoperative outcomes stratified by manometric thresholds

ROC analysis demonstrated that percent failed contractions had limited ability to discriminate worse long-term postoperative symptom outcomes (Supplemental Fig. 5). Across symptom domains, AUC values were approximately 0.58–0.60, suggesting poor discriminatory performance. These findings further support that conventional manometric thresholds alone were limited predictors of long-term symptom burden in this cohort.

### Multivariate analysis of long-term symptom scores

We performed an adjusted linear regression model including ETT group, baseline symptom severity, age, BMI, and hiatal hernia type. We found that ETT ≥ 5.375 s remained significantly associated with higher long-term RSI and GCSI scores. Specifically, ETT ≥ 5.375 s was associated with a 5.61-point higher long-term RSI score (95% CI 0.84 to 10.38, *p* = 0.021) and a 5.09-point higher long-term GCSI score (95% CI 1.51 to 8.67, *p* = 0.005). Associations with GERD–HRQL (β = 2.32, 95% CI −1.49 to 6.13, *p* = 0.233) and Eckardt score (β = 0.81, 95% CI −0.24 to 1.86, *p* = 0.131) were not statistically significant after adjustment. These findings suggest that prolonged ETT is independently associated with selected domains of long-term symptom burden (Supplemental Table 7).

## Discussion

In this retrospective cohort of patients undergoing antireflux surgery, preoperative ETT was associated with several postoperative symptom outcomes and correlated with select manometric measures of esophageal function. Longer ETT was associated with higher percent failed contractions and lower average distal esophageal contraction amplitude on preoperative manometry, and patients with longer preoperative ETT demonstrated greater symptom burden at later postoperative follow-up across several patient-reported outcome measures.

These findings are clinically relevant because current GERD guidelines continue to emphasize upper endoscopy, manometry, and pH testing in the preoperative evaluation of medically refractory reflux, while also acknowledging ongoing uncertainty regarding the strength of evidence supporting specific components of that workup [9]. Concurrently, the ACG guideline states that barium esophagram should not be used solely as a diagnostic test for GERD [29]. Our goal was therefore not to suggest esophagram as a replacement diagnostic test for GERD, but rather to study if an esophagram-derived metric (ETT) could serve as an adjunctive screening tool during preoperative assessment. Given the lack of consensus regarding which preoperative tests are necessary, we sought to determine whether a normal ETT on esophagram might help identify selected patients in whom routine manometry could be deferred.

From a clinical standpoint, our findings suggest that ETT may help identify patients in whom normal esophageal transit makes severe underlying dysmotility less likely. Because the negative predictive value of ETT < 5.375 s was relatively high across symptom domains, a normal ETT may have value as a screening marker to help determine when routine preoperative manometry might be safely deprioritized in selected patients, particularly when the esophagram is otherwise normal and there is no clinical concern for a major motility disorder [30]. This idea is consistent with prior studies that critique the universal necessity of manometry before antireflux or hiatal hernia surgery, particularly when other elements of the preoperative evaluation are reassuring [12, 13, 17]. Importantly, our data does not support disuse of preoperative manometry altogether, since it remains critical for exclusion of achalasia and other important motility disorders.

An important finding of this study is that the relationship between ETT and outcomes was more apparent at longer-term follow-up than in the early postoperative period. This pattern may indicate that delayed esophageal clearance has greater relevance to persistent or recurrent symptom burden over time than to short-term postoperative recovery. The separation observed in later Eckardt, GCSI, RSI, and GERD–HRQL trajectories suggests that ETT may capture an aspect of esophageal function that is not fully reflected by static anatomic findings alone. This longer-term association distinguishes our study from prior work that has focused more on early postoperative dysphagia or immediate perioperative outcomes and represents a strength of our analysis [16, 22].

The ROC analyses identified 5.375 s as the threshold used for subsequent analyses because it performed consistently across multiple symptom domains. Even so, the predictive performance of ETT as a standalone test was modest. Specificity and positive predictive values were limited, and the likelihood ratios were not strong enough to support ETT as an independent rule-in test for poor postoperative outcome. In contrast, the higher negative predictive values suggest that shorter ETT may be more useful in identifying patients less likely to experience poor long-term symptom burden. Moreover, ETT was more associated with long-term patient-reported symptom burden rather than with harder downstream events such as dilation, repeat imaging, or reoperation. This likely reflects the multifactorial nature of reintervention after foregut surgery and the low event rate in our cohort. Accordingly, ETT may be more useful as a tool for counseling patients about expected symptom trajectories than as a predictor of procedural failure requiring additional intervention.

The correlation between longer ETT and higher percent failed contractions supports the physiologic relationship between delayed fluoroscopic transit and impaired esophageal clearance. The inverse correlation between ETT and distal contraction amplitude further suggests that prolonged transit may reflect weaker distal esophageal body function. Although these correlations were modest, they provide internal consistency between the radiographic and manometric datasets and support the concept that ETT may function as a convenient and cost-effective fluoroscopic marker of esophageal motility.

The manometry analyses in this cohort were less robust than the ETT analyses, but these findings should be interpreted in context. Only a subset of patients had manometry available, and abnormal manometric subgroups were small for several variables. In addition, patients with achalasia and re-operative foregut surgery were excluded, narrowing the physiologic range of motility abnormalities in the final cohort. As a result, the study may have been underpowered to detect clinically meaningful associations between several conventional manometric thresholds and postoperative outcomes. The weaker performance of manometric thresholds in this analysis therefore should not be interpreted that manometry lacks clinical value, particularly when exclusion of major motility disorders before ARS remains the primary purpose of manometry.

A substantial proportion of potentially eligible esophagram studies, especially from outside institutions, could not be analyzed because of insufficient images, which ultimately limited the statistical power of the study but also highlights an important limitation of current esophagram practice: lack of standardized acquisition for motility assessment. These findings emphasize its usefulness depends heavily on how the study is performed and recorded. If ETT is to be used more broadly in preoperative risk stratification, standardized acquisition and interpretation protocols will be necessary. This is an area of future study and validation.

In our adjusted multivariable model accounting for baseline symptom severity, age, BMI, hiatal hernia type, and prolonged ETT remained significantly associated with higher long-term RSI and GCSI scores, while associations with GERD–HRQL and Eckardt score were attenuated. These findings suggest that ETT may capture a physiologic component of postoperative symptom burden, particularly extra-esophageal/reflux-related and nausea/vomiting/gastroparesis-type symptoms, but does not uniformly predict all long-term outcome domains. Therefore, ETT should be interpreted as an adjunctive risk-stratification marker rather than a standalone predictor of postoperative outcomes.

Several additional limitations should be considered. First, this was a retrospective, single-center study and is therefore subject to inherent selection bias, incomplete data capture, and heterogeneity in clinical decision-making. Second, there was loss to follow-up in postoperative HRQL data over time, which necessitated combining the 3- and 5-year follow-up windows and using the furthest available time point for each patient. Although this approach preserved longer-term data, pooling scores beyond 3 years may have introduced heterogeneity in follow-up timing and influenced outcomes if the distribution of 3– and 5–year data was unequal.

Third, normative thoracic ETT thresholds are not well established, and the cutoff used in this study was derived from internal ROC analyses rather than external validation. While this represents a reasonable threshold based on manometric studies defining normal distal latency (DL) as < 8.8 s from pharyngeal swallow to the LES using the 95th percentile [31] and considering that the thoracic esophagus comprises approximately 80% of total esophageal length, the lack of external validation limits generalizability and introduces a risk of overfitting. Additionally, fluoroscopic transit measurements are technique-dependent and may be influenced by factors such as contrast volume, patient positioning, frame rate, and reader interpretation. This study did not assess the interobserver variability in the ETT measurement, which will be addressed in a future study to assess the reproducibility of this measurement.

In summary, these limitations suggest potential challenges regarding generalizability and applicability in routine clinical practice without prospective validation. The specificity and modest likelihood ratios also limit the clinical utility of ETT as a predictive tool, underscoring the need for further investigation. Additionally, our multivariable analysis is limited by the modest complete-case sample size and the low explanatory power of the overall models, with adjusted R^2^ values indicating that much of the variation in long-term symptom scores remains unexplained by ETT, baseline symptom severity, age, BMI, and hiatal hernia type. Finally, postoperative symptom questionnaires are inherently patient-reported and may be influenced by factors beyond esophageal physiology alone.

Despite these limitations, this study supports further investigation of ETT as a simple and potentially useful component of the preoperative workup for antireflux surgery. We hope our findings can help create a template for standardizing how UGIs are performed. Esophagram is widely available, less invasive than manometry, and already provides important anatomic information. If standardized and prospectively validated, ETT may provide additional functional information to help identify patients at higher or lower risk of persistent postoperative symptoms.

## Electronic supplementary material

Below is the link to the electronic supplementary material.

Supplementary file1 (DOCX 1527 kb)

## Appendix

### Appendix Item 1: Detailed GERD–HRQL, RSI, GCSI, and Eckardt score questionnaires

GERD health-related quality of life questionnaire—GERD–HRQL Questions:

1. How bad is your heartburn?
2. Do you have heartburn when lying down?
3. Do you have heartburn when standing up?
4. Do you have heartburn after meals?
5. Does your heartburn change your diet?
6. Does your heartburn wake you from sleep?
7. Do you have difficulty swallowing?
8. Do you have pain with swallowing?
9. If you take medication, does this affect your daily life?

Score Range:

0 = no symptoms.

1 = Symptoms noticeable, but not bothersome.

2 = Symptoms noticeable and bothersome, but not everyday.

3 = Symptoms bothersome everyday.

4 = Symptoms affect daily activities.

5 = Symptoms are incapacitating; unable to do daily activities.

Total SCORE = 0–45.

Reflux symptom index—RSI Questions:

1. Hoarseness or a problem with your voice
2. Clearing your throat
3. Excess throat mucous or postnasal drip
4. Coughing after you eat or after lying down
5. Breathing difficulties or choking episodes
6. Troublesome or annoying cough
7. Sensations of something sticking in your throat
8. How bad is your heartburn?
9. Do you have difficulty swallowing?

Score:

0 = no symptoms.

1 = Symptoms very mild.

2 = Symptoms mild.

3 = Symptoms moderate.

4 = Symptoms severe.

5 = Symptoms very severe.

SCORE total = 45.

Gastroparesis Cardinal Symptom Index—GCSI Questions: 1. nausea.

2. Bloating
3. Loss of appetite
4. Stomach fullness
5. Feel excessively full after meals
6. Struggle finishing a normal sized meal
7. Retching
8. Vomiting
9. Stomach visibly larger

Score Range:

0 = no symptoms.

1 = Symptoms very mild.

2 = Symptoms mild.

3 = Symptoms moderate.

4 = Symptoms severe.

5 = Symptoms very severe.

TOTAL = 45.

Eckardt Score Questions:

1. Difficulty swallowing
2. Chest pain (score above)
3. Regurgitation (score above)
4. Weight loss in past year
5. 0 = none
6. 1 = 1–11 lbs
7. 2 = 11–22 lbs
8. 3 = more than 22lbs

Score Range.

0 = none.

1 = occasional.

2 = Daily.

3 = Each Meal.

Total SCORE = 0–12.

### Appendix Item 2

See Table 2.

**Table 2:** STROBE statement—checklist of items that should be included in reports of cohort studies

	Item No	Recommendation	Page No	Relevant text from manuscript
Title and abstract	1(a)	Indicate the study’s design with a commonly used term in the title or the abstract	1–2	Title page identifies the article type as 'Observational study, retrospective cohort,' and the abstract Methods begins 'In this retrospective cohort study…'
	1(b)	Provide in the abstract an informative and balanced summary of what was done and what was found	2	Abstract includes background, cohort size (n = 174; manometry subset n = 92), study timeframe, blinded esophagram review, ROC/correlation findings, and cautious interpretation
Introduction	2	Explain the scientific background and rationale for the investigation being reported	3	Introduction reviews GERD burden, role of antireflux surgery, uncertainty in preoperative workup, limitations of routine manometry, and rationale for evaluating esophagramderived ETT
	3	State specific objectives, including any prespecified hypotheses	3–4	The study objective is to determine whether ETT correlates with HRM findings and predicts postoperative outcomes after ARS, with the aim of informing preoperative decision-making
Methods	4	Present key elements of study design early in the paper	1, 4	Article type is listed on the title page, and Methods states that this is a retrospective cohort study using a prospectively maintained database
	5	Describe the setting, locations, and relevant dates, including periods of recruitment, exposure, follow-up, and data collection	2, 4	Single tertiary academic center at the University of Wisconsin; operations from 2014–2025; follow-up at 6 weeks, 1 year, 3 years, and 5 years; 3- and 5-year assessments were combined using the most distant available follow-up
	6(a)	Give the eligibility criteria, and the sources and methods of selection of participants. Describe methods of follow-up	4–5	Adult patients undergoing Nissen or Toupet fundoplication, with or without concurrent hiatal hernia repair, were eligible if they had a preoperative esophagram and at least 1 postoperative HRQL assessment. Exclusions included achalasia and previous operative foregut surgery
	6(b)	For matched studies, give matching criteria and number of exposed and unexposed	N/A	Not a matched cohort study
	7	Clearly define all outcomes, exposures, predictors, potential confounders, and effect modifiers. Give diagnostic criteria, if applicable	4–5	Exposure/predictor: preoperative ETT Outcomes: postoperative GERD-HRQL, Eckardt, GCSI, RSI, and reinterventions. Manometric variables were analyzed as physiologic comparison measures. Achalasia was an exclusion diagnosis
	8*	For each variable of interest, give sources of data and details of methods of assessment (measurement). Describe comparability of assessment methods if there is more than one group	4–5	Clinical data were abstracted from a prospectively maintained database and REDCap. HRQL instruments are described. Preoperative esophagrams were re-reviewed by radiologists blinded to outcomes, and ETT was calculated from DICOM frame counts and frame rate
	9	Describe any efforts to address potential sources of bias	4–5, 910	Radiologists were blinded to patient information and outcomes. Patients without interpretable motility imaging were excluded. The discussion acknowledges single-center retrospective design, selection bias, incomplete data capture, and technique dependence of fluoroscopic measurements
	10	Explain how the study size was arrived at	4–5	All eligible adult patients in the institutional database from 2014–2025 who met inclusion criteria. No a priori sample size calculation was performed
	11	Explain how quantitative variables were handled in the analyses. If applicable, describe which groupings were chosen and why	5–8	ETT was analyzed continuously in correlation analyses and dichotomized at 5.375 s for subsequent analyses because this threshold performed consistently across multiple symptom domains. Symptom thresholds and manometric thresholds are reported where used
	12(a)	Describe all statistical methods, including those used to control for confounding	5–6	Analyses were performed in a Julius AI-assisted Python notebook environment with author and statistician review. Reported methods include Welch t tests for continuous group comparisons, chi-square tests for categorical baseline comparisons, Pearson correlations with 95% CIs, ROC/AUC analyses with Youden index for exploratory cutoff selection, and Mann–Whitney U testing for reintervention analyses. No multivariable adjustment was performed
	12(b)	Describe any methods used to examine subgroups and interactions	5–8	Subgroup/comparative analyses included the manometry subset (n = 92), ETT-stratified comparisons (< 5.375 s vs > = 5.375 s), and exploratory ROC analyses for manometric thresholds. No formal interaction modeling was performed
	12(c)	Explain how missing data were addressed	4–5	Missing data were handled by exclusion only. No imputation was performed because the number of missing data points was too large to support mean or median imputation
	12(d)	If applicable, explain how loss to follow-up was addressed	4, 9–10	Follow-up availability decreased over time; therefore, 3- and 5-year HRQL time points were combined, and the most distant available assessment was used for each patient. This limitation is acknowledged in the Discussion
	12(e)	Describe any sensitivity analyses	6–8	Additional exploratory analyses are reported, including alternative ROC cutoffs, reintervention analyses, and manometry threshold analyses; however, no formal prespecified sensitivity analysis was performed
Results	13(a)	Report numbers of individuals at each stage of study—eg numbers potentially eligible, examined for eligibility, confirmed eligible, included in the study, completing follow-up, and analysed	5	Results and Fig. 1 report 367 potentially eligible patients, exclusions for outside/inadequate imaging and in-house studies lacking usable motility imaging, and a final analytic cohort of 174, including a manometry subset of 92
	13(b)	Give reasons for nonparticipation at each stage	5	Reasons for exclusion include outside institution studies lacking appropriate functional motility imaging, missing motility data, non-visualization of the LES, premature fluoroscopic termination, and late cine start
	13(c)	Consider use of a flow diagram	5	Yes. Figure 1 presents the cohort selection flow diagram
	14(a)	Give characteristics of study participants (eg demographic, clinical, social) and information on exposures and potential confounders	5, 7–8	Baseline age, BMI, sex, race, presenting symptoms, operative approach, fundoplication type, and hiatal hernia characteristics are reported in Table 1 and Supplemental Figure 3, including ETT-stratified baseline comparisons
	14(b)	Indicate number of participants with missing data for each variable of interest	5, 7–8	The manuscript describes exclusions due to missing or unusable motility imaging, but it does not provide a variable-by-variable missing data count for every reported variable
	14(c)	Summarize follow-up time (eg, average and total amount)	4, 6, 9	Follow-up windows are reported at 6 weeks, 1 year, 3 years, and 5 years, and long-term outcomes use the furthest available 3- or 5-year assessment. Average or total follow-up time is not separately summarized
	15*	Report numbers of outcome events or summary measures over time	6–8	Longitudinal symptom outcomes are reported across baseline, 6-week, 1-year, and combined 3– to 5–year time points; reintervention outcomes and manometry-based analyses are also summarized
	16(a)	Give unadjusted estimates and, if	6–8	Unadjusted correlations, AUCs, p values, 95% CIs, sensitivity/specificity, PPV/NPV, and
		applicable, confounder adjusted estimates and their precision (eg, 95% confidence interval). Make clear which confounders were adjusted for and why they were included		likelihood ratios are reported. No confounder adjusted analyses were performed
	16(b)	Report category boundaries when continuous variables were categorized	6–8	Category boundaries are reported, including ETT < 5.375 s versus > = 5.375 s and manometric percent-failed-contraction cutoffs derived from ROC analysis
	16(c)	If relevant, consider translating estimates of relative risk into absolute risk for a meaningful time period	N/A	Not applicable; no relative-risk model was used
	17	Report other analyses done—eg analyses of subgroups and interactions, and sensitivity analyses	6–8	Other analyses include the manometry subset correlations, diagnostic test characteristics comparing manometry and esophagram, reintervention analyses, and ROC analyses of manometric thresholds. No multiple comparison correction was performed
Discussion	18	Summarise key results with reference to study objectives	8–10	The Discussion restates that preoperative ETT correlated with select manometric measures and was associated with greater long-term postoperative symptom burden, directly addressing the study objectives
	19	Discuss limitations of the study, taking into account sources of potential bias or imprecision. Discuss both direction and magnitude of any potential bias	9–10	Limitations include retrospective single-center design, selection bias, incomplete data capture, decreasing HRQL follow-up, internally derived cutoff, technique dependence of fluoroscopy, and patient-reported outcomes
	20	Give a cautious overall interpretation of results considering objectives, limitations, multiplicity of analyses, results from similar studies, and other relevant evidence	8–10	Interpretation frames ETT as an adjunctive screening and risk-stratification tool rather than a replacement for manometry; findings are compared with the current guidelines and prior literature, and the exploratory nature of the analyses is acknowledged
	21	Discuss the generalisability (external validity) of the study results	10	The manuscript notes the single-center design and the need for standardized acquisition and prospective validation before broader application, appropriately limiting generalizability claims
Other information	22	Give the source of funding and the role of the funders for the present study and, if applicable, for the original study on which the present article is based	1	Funded through the Department of Minimally Invasive Surgery at the University of Wisconsin-Madison, with Anne Lidor, MD, MPH, as principal investigator
