# Supplementary material for: Predictive value of preoperative esophagram in postoperative outcomes after antireflux surgery
Source: Surg Endosc. 2026 Jul 31;40(10):9000–14. doi: 10.1007/s00464-026-13126-4 (PMC13633291; doi:10.1007/s00464-026-13126-4)
Supplement: Supplementary file 1 [file 464_2026_13126_MOESM1_ESM.docx]

**Supplemental Figure Legends**

Supplemental Figure 1. Fluoroscopic motility study with ETT measurement. Patients are in a prone oblique position for the dynamic motility series, which removes gravity assist. Swallowed barium contrast appears as dark signal in the esophagus, stomach, and small bowel. Two representative images from the dynamic series are shown. Frame number was recorded when the inverted “V” of contrast bolus reaches the first rib designed as ‘inlet’ (a) and when it reached the lower esophageal sphincter (b). The difference in frame number converted by the frame rate gives the ETT.

Supplemental Figure 2. Pairwise correlation heat map and selected regression analyses of ETT, manometric variables, and symptom scores. The heat map displays pairwise correlation coefficients across baseline, short-term, and long-term postoperative symptom scores together with ETT and manometric variables. The accompanying table summarizes selected regression analyses highlighting associations of interest between ETT, manometric measures, and symptom outcomes. The heat map displays exploratory pairwise Pearson correlation coefficients across continuous variables. The accompanying table lists unadjusted pairwise regressions/correlations with N, r, R², p values, and approximate 95% confidence intervals from the original analysis. No multiple-comparison correction was performed.

Supplemental Figure 3. Baseline demographic, clinical, and operative characteristics stratified by esophageal transit time. Patients were grouped by preoperative ETT using a threshold of 5.375 seconds. Demographic variables, presenting symptoms, and operative characteristics were largely similar between groups, except for dyspnea and hiatal hernia type distribution, which differed significantly. Values are presented as mean ± standard deviation or number (%). P values compare patients with ETT ≥5.375 seconds versus ETT <5.375 seconds.

Supplemental Figure 4. Preoperative esophageal transit time and postoperative reintervention. Mean preoperative ETT is compared between patients who did and did not undergo postoperative reintervention, including any reintervention, endoscopic dilation, reoperation, or repeat imaging. Mean ETT values are shown as mean ± SEM by reintervention status. Between-group comparisons were performed with two-sided Mann–Whitney U tests; no statistically significant associations were identified. 95% confidence intervals for mean differences were not calculated in the original analysis.

Supplemental Figure 5. Receiver operating characteristic analysis of percent failed contractions for long-term-symptom outcomes. ROC curves demonstrate the performance of percent failed contractions on manometry for predicting worse symptom outcomes more than 3 years after surgery across GERD, NV, OS, and Eckardt domains. Discriminatory ability was limited across all outcomes, with AUC values of 0.599 for >3-year GERD score, 0.599 for >3-year Eckardt score, 0.591 for >3-year GCSI score, and 0.579 for >3-year RSI score. The accompanying table summarizes sample size, case distribution, optimal cutoff values by Youden index, sensitivity, specificity, and Youden index for each outcome. The average optimal cutoff across outcome variables was 10.25% failed contractions.

Supplemental Table 6. Normality testing results for selected continuous variables, including sample size, mean ± SD, median [IQR], Shapiro-Wilk p value, and interpretation.

Supplemental Table 7. Adjusted robust linear regression results for the association between ETT ≥5.375 seconds and long-term postoperative symptom scores, including model N, β coefficient, robust 95% confidence interval, p value, and adjusted R².
